# Maxillary Dentigerous Cyst with Double Wolf Teeth in a 3-Year-Old Quarter Horse Mare

**DOI:** 10.1155/2021/5532236

**Published:** 2021-10-07

**Authors:** Constanze Gutzmer, Pieter Nijdam

**Affiliations:** ^1^Pferdeklinik am Kottenforst, Germany; ^2^Tierklinik Schneichel, Germany

## Abstract

Dentigerous cysts are characterised by the formation of cysts containing dental material with a variable level of development. They are the result of a distinct embryological phenomenon. Usually, they are of significant clinical relevance in horses, especially in tandem with ectopic tooth. Contrarily, supernumerary teeth or typical polyodontias usually have limited impact. In this case report, we describe the occurrence of a supernumerary first premolar (Triadan 105). Dissimilar to known scientific literature however was the formation of a cystic structure around the supernumerary tooth. Surgical removal of the dentigerous cyst is discussed, as are the possible causes for the formation of the cystic structure. Based on this case report, we suspect that the formation of a cystic structure may not be limited to atypical polyodontias, as they may also occur in supernumerary teeth.

## 1. Introduction

Dentigerous cysts or atypical polyodontias are a result of dental dysgenesis. They are described as epithelium-lined cystic structures in soft tissues or bone, which develop out of cellular debris of the Hertwig's epithelial root sheath. These cells are part of the enamel organ, which are a precursor of the later origin of the tooth roots. Up to now, the formation of cysts has only been related to ectopic dental tissues [1]. Because of failure of the first branchial cleft to close during embryonic development, tooth material can, for instance, be found in the region of the ear or the maxilla. Often, this tooth material is surrounded by fluid, produced by the fine membrane of the cyst [2].

Supernumerary teeth or typical polyodontias are not usually described with concurrent formation of a cystic structure around it. Usually, they refer solely to the development of a supernumerary tooth, often of minor clinical relevance. Irregular shapes or rotation of supernumerary teeth may sometimes be observed [1]. The first premolar or wolf tooth can also vary greatly in size and shape. Position and occurrence of eruption may also vary. However, supernumerary wolf teeth are seldomly diagnosed [3]. To the author's knowledge, supernumerary wolf teeth encapsulated in a cystic structure have not been described yet.

## 2. Case Presentation

A 3-year-old Quarter Horse mare was presented to the equine clinic am Kottenforst. Reasons for presentation were clear signs of pain while grooming the right side of the head. The mare was being broken in at the time, and the owner reported a fending reaction when grooming or applying the bit, specifically at the right side of the head. Increased salivation was also observed. No clinical examination or routine dental treatment had been performed before presentation.

The clinical examination of the mare was uneventful, although the mare did clearly avoid palpation of the right rostral maxillary region. An examination of the oral cavity was performed under sedation. The gingiva rostral of the right maxillary second premolar (Triadan 106) was clearly hyperemic. On palpation, the gingiva in this area seemed hard and distended. The first premolar teeth were not visible at the time of examination. No further abnormalities were discovered in the oral cavity.

**Figure 1:**
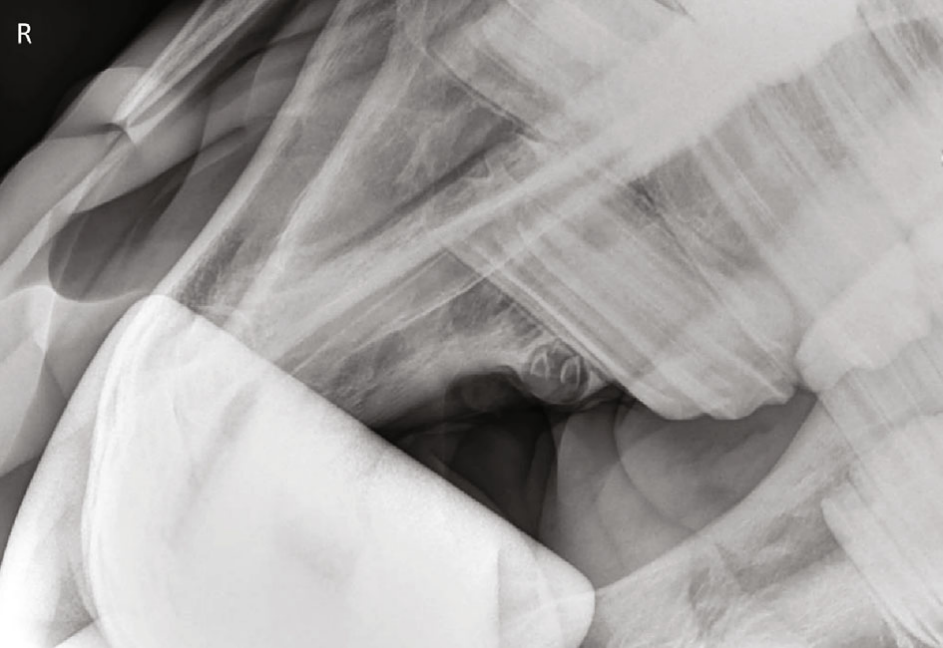
Radiograph (70°/-15°; OM) of the right rostral maxilla using a Castell's mouth gag. Two distinct dental structures are visible, surrounded by a cystic capsule.

**Figure 2:**
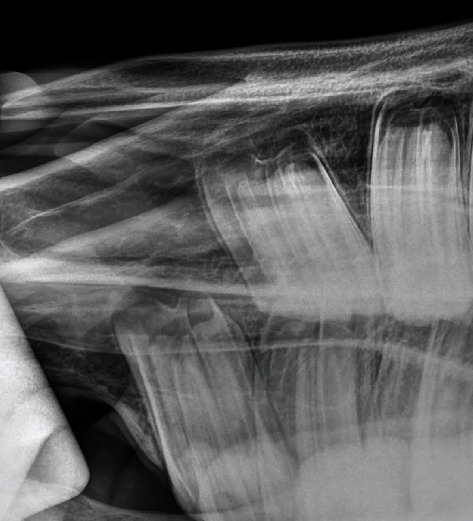
Radiograph (70°/-15°; OM) of the left rostral maxilla using a Castell's mouth gag shows no signs of a wolf tooth.

A radiographic examination was performed of the right rostral maxillary region and of the left side for comparison. The radiographic examination is based on the hemisphere model [4]. A right and left dorsocranial to dorsocaudal 70°/-15°, OM centered on the first premolar was prepared using a Castell's mouth gag. On the radiograph of the right side a round, a radiolucent structure with two distinct radiopaque structures within was visible (Figure 1). The radiolucent structure was completely enclosed in the maxilla. The radiograph of the left side showed no radiographic abnormalities (Figure 2). Based on imaging and clinical examination, a supernumerary first premolar or wolf tooth (Triadan 105) with concurrent cyst formation was diagnosed.

**Figure 3:**
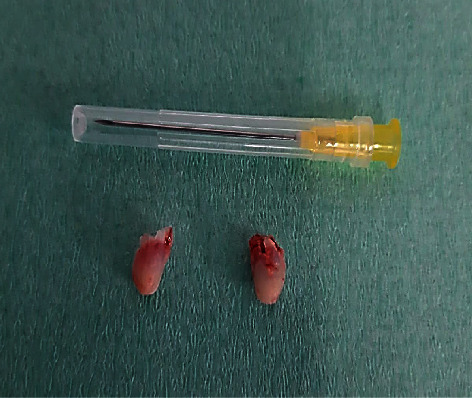
Two incomplete tooth structures after removal out of the cyst. Size comparison with a 40 mm 20 G needle.

**Figure 4:**
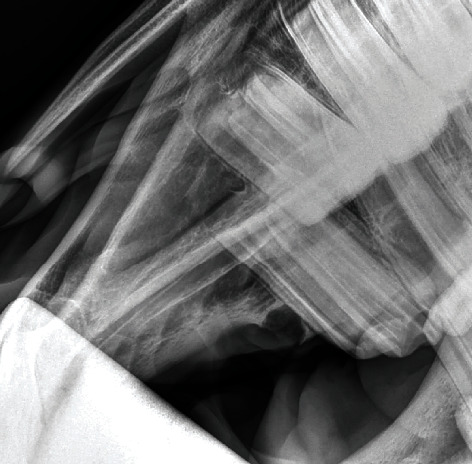
Radiograph (70°/-15°; OM) of the right rostral maxilla using a Castell's mouth gag after complete removal of the cyst and the two tooth-like structures.

Treatment consisted of surgical removal under standing sedation. After placing an in-dwelling catheter in the left jugular vein, the mare was sedated with detomidine hydrochloride (0.01 mg/kg) and butorphanol (0.05 mg/kg) as well as romifidine (0.04 mg/kg). A block of the right N. infraorbitalis was performed with 3 ml of lidocaine 2%; the gingiva in the affected area was also infiltrated with 10 ml lidocaine 2%. An incision of 1.5 cm was made over the distended gingiva. To enter and open the cyst, the maxillary bone was removed by means of an osteotome. A very thin (0.1 cm) membrane was lining the cyst. After opening, approximately 1 ml of milky-white to yellowish mucous fluid drained out of it. Two small dental structures were removed from this cavity afterwards (Figure 3). The fluid and the membrane were submitted for histopathological examination. The complete cystic structure was subsequently removed after which curettage and lavage of the affected area was performed. Complete excision was confirmed radiographically (Figure 4).

The resulting cavity was filled up with iodine gauzes and honey cream (Jodotamp®, Mielosan®). There was no connection identified between the cavity and the nearby alveoli of the second premolar (Triadan 106).

Postoperatively, the mare showed no signs of discomfort and feed intake was normal. She was treated with flunixin meglumin (1.1 mg/kg q24h i.v.) for 3 days postoperatively. The tamponade was replaced every second day for 8 days; afterwards, the cavity was flushed daily with saline solution. Three days postoperatively, the mare was discharged from the equine hospital. At check-up 14 days postoperatively, the cavity was almost fully filled with granulation tissue. The owner did not observe any pain or excess salivation after the surgical treatment.

A cytological examination was performed of the fluid contents of the cavity and of the inner membrane of the cyst. The fluid was classified as transudate; few mesothelial cells and neutrophils were also seen. In the membrane lining, the cavity epithelial and goblet cells were identified. The two tooth structures were not examined separately. Macroscopically, both teeth were presented with a crown and a neck but had no inner filling. Roots however were not present, and there were no signs of pulp tissue.

## 3. Discussion

This case report describes the diagnosis and surgical treatment of a dentigerous cyst concurrent to a supernumerary first premolar or wolf tooth in a 3-year-old Quarter Horse mare.

In this case, it is presumed that one of the two teeth in the cyst was a supernumerary first premolar or wolf tooth (Triadan 105), a so-called typical polyodontia. The region where both teeth were found is typical and has been described several times in scientific literature [5]. Still, the concurrent formation of a cyst filled with fluid encapsulating these teeth is uncommon for a typical polyodontia. This is usually observed in atypical polyodontias, especially around the ear region. Often, the dentigerous cyst is characterised by the formation of draining tracts [6].

In our case, however, a draining tract was not observed. It is still possible that a draining tract might have developed in the future and the increasing sensitivity on palpation and grooming may support this. One study on supernumerary teeth and dentigerous cysts reported a mean age at presentation of 3 years, like our patient. Over half of the patients reported draining tracts in connection with an ectopic tooth, but cases were also described in which only a swelling or irritation developed at the base of the ear [7]. In our case, we cannot exclude the possibility that a draining tract might have developed later.

The two tooth-like structures within the cyst were not fully developed. There could be a possible connection between the incomplete development of the teeth and the development of the cyst. After the enamel organ preformed the tooth roots, dental sacs develop. Within these dental sacs, cells from the epithelial root sheath often remain behind. In case of inflammation because of doubled expression of 105, these cells can cause cyst proliferation [8]. In line with this is the development of cysts around ectopic teeth. However, contrary to the case described, ectopic tooth formation is based on expression of additional molars out of the maxillary bulges in a caudal direction [7]. While ectopic teeth only express caudally, the presented case shows a cystic formation in the premolar region.

The prognosis for surgical excision of a dentigerous cyst relies mainly upon the anatomical position and whether the structure can be removed to a full extent or not [9]. In the described case, the cyst did not involve any vascular or nervous structures. During these surgical interventions, especially hemorrhage or iatrogenic damage to nerves and vessels may negatively impact prognosis [6]. In the current case, no complications occurred.

The cystic structure was opened by using an osteotome, and the membrane lining the cavity was removed by means of curettage and subsequent lavage. Should epithelial cells remain, a new membrane could reform and complaints could recur. In this case, however, no such complications were observed.

To the authors' knowledge, this is the first report describing a supernumerary wolf tooth in a cystic structure. Based on our findings, we suspect that the formation of cystic structures may not only occur related to ectopic teeth such as an atypical polyodontia but may also occur in some forms of supernumerary teeth or typical polyodontias. Further research is warranted to confirm this suspicion.

## Data Availability

The data used to support the findings of this study are included within the article.
